# Epigenetic Abnormalities in Chondrosarcoma

**DOI:** 10.3390/ijms24054539

**Published:** 2023-02-25

**Authors:** Michał Bereza, Mateusz Dembiński, Agnieszka E. Zając, Jakub Piątkowski, Monika Dudzisz-Śledź, Piotr Rutkowski, Anna M. Czarnecka

**Affiliations:** 1 Department of Soft Tissue/Bone Sarcoma and Melanoma, Maria Sklodowska-Curie National Research Institute of Oncology, 02-781 Warsaw, Poland; 2 Faculty of Medicine, Medical University of Warsaw, 02-091 Warsaw, Poland; 3 Institute of Genetics and Biotechnology, Faculty of Biology, University of Warsaw, 02-106 Warsaw, Poland; 4 Department of Experimental Pharmacology, Mossakowski Medical Research Centre Polish Academy of Sciences, 02-106 Warsaw, Poland

**Keywords:** chondrosarcoma, epigenetic mechanisms, targeted therapy

## Abstract

In recent years, our understanding of the epigenetic mechanisms involved in tumor pathology has improved greatly. DNA and histone modifications, such as methylation, demethylation, acetylation, and deacetylation, can lead to the up-regulation of oncogenic genes, as well as the suppression of tumor suppressor genes. Gene expression can also be modified on a post-transcriptional level by microRNAs that contribute to carcinogenesis. The role of these modifications has been already described in many tumors, e.g., colorectal, breast, and prostate cancers. These mechanisms have also begun to be investigated in less common tumors, such as sarcomas. Chondrosarcoma (CS) is a rare type of tumor that belongs to sarcomas and is the second most common malignant bone tumor after osteosarcoma. Due to unknown pathogenesis and resistance to chemo- and radiotherapies of these tumors, there is a need to develop new potential therapies against CS. In this review, we summarize current knowledge on the influence of epigenetic alterations in the pathogenesis of CS by discussing potential candidates for future therapies. We also emphasize ongoing clinical trials that use drugs targeting epigenetic modifications in CS treatment.

## 1. Introduction

Chondrosarcoma (CS) is a heterogeneous type of primary bone tumor that presents different morphologic features and responses to treatment. CS constitutes the second most common primary solid bone tumor following osteosarcoma [1]. According to the most recent WHO classification [2], CS can be classified into primary central CS, secondary central CS, and secondary peripheral CS (grade 1, and grade 2 and 3), which had previously been described as conventional CS, periosteal, dedifferentiated, and mesenchymal CS. The most common type of this tumor is primary central CS (75% of cases). The risk of metastases depends on the grade of CS. Low-grade CS (grade 1) has about a 10% risk of metastases, whereas in high-grade CS (grades 2 and 3) the risk is 50–70% [3]. The long bones and pelvic bones are the most commonly affected [4]. Patients usually complain of long-lasting pain and swelling close to the altered bone [5]. Tissue biopsies and imaging studies are essential to diagnose and differentiate CS from other tumors [5]. The most efficient method of treating these tumors is surgical excision. Other therapeutic options, such as chemotherapy/radiation therapies, are not effective in the treatment of CS (except for the use of chemotherapy in dedifferentiated CS containing high-grade spindle cell components and mesenchymal CS, and for the use of radiation therapy after incomplete resection or local recurrence in intermediate CS and high-risk CS) [5,6]. Due to the limited therapeutic options in CS treatment, it is essential to look for new methods of treatment. Therefore, focusing on epigenetic mechanisms may be promising in the development of new therapeutic methods. These mechanisms include e.g., DNA methylation and demethylation, histone modifications, and microRNA-regulated epigenetic changes [7,8]. Epigenetic modifications might be considered as potential targets for specific drugs, as well as diagnostic and prognostic factors [9]. In this review, we discuss the current state of knowledge of epigenetics in CS, describe novel potential therapeutic targets, and summarize ongoing epigenetics-based clinical trials.

## 2. DNA Methylation in Chondrosarcoma

### 2.1. Hypomethylation of DNA

DNA methylation is a process of adding a methyl group to a nucleotide base of the DNA, catalyzed by the action of DNA methyltransferase enzymes (DNMTs) [10,11]. DNA hypomethylation, in contrast to DNA methylation, refers to the loss of the methyl group(CH_3_) in the 5-methylcytosine nucleotide [12]. Two DNMT inhibitors, 5-azacytidine (azacytidine) and 5-aza-2′-Deoxycytidine (decitabine, DAC), have been already approved by the FDA for the treatment of some hematological diseases e.g., acute myeloid leukemia (AML) and myelodysplastic syndrome (MDS) [13]. Hypomethylation can be divided into two classes: global hypomethylation and hypomethylation of a single gene [10]. Among global hypomethylation, we can distinguish repetitive DNA sequences, such as Satellite 1 and long interspersed nuclear elements 1 (LINE-1; L1). Satellite 1, a member of the satellite DNA family (mentioned in Table 1), has been associated with a variety of important cell functions, from correct segregation of chromosomes and genome stability to its association with regulatory functions through satellite transcripts [14]. L1 (Table 1) plays a role in genome instability in tumors and, via its retrotransposition activity, participates in tumor progression [15]. Therefore, both Satellite 1 and L1 may become promising biomarkers and/or therapeutic targets. In the study by Hamm et al. [16], performed on Swarm rat CS (SRC) cells treated with DAC, both repetitive DNA sequences (Satellite 1 and L1) were hypomethylated after treatment with DAC. In the same study, SRY-related HMG-box transcription factor 2 (Sox-2) and neurite outgrowth-promoting factor 2 (midkine, MDK) were found to be overexpressed and there was a decrease in the methylation level in the promoter of both genes (*SOX2* and *MDK*) after DAC treatment (Table 1) [16]. After DAC administration, the tumor became more invasive, grew faster, and was larger, both in in vitro and in vivo models [16]. However, the effect of DAC is different among various CS cell lines. The research mentioned above was carried out on the SRC-MSCV3-LTC cell line, while Bui et al. [17] have shown in the H-EMC-SS cell line that DAC can restrict CS cell growth and invasiveness through increased expression of the Heparin-Glucosamine 3-O-Sulfotransferase (*3-OST-2*) gene (Table 1). Research conducted on SRC cells revealed that Sox-2 and MDK may have a significant impact on the pathogenesis of CS [16]. In general DAC is effective in the treatment of hematopoietic malignancies; however, it can also promote tumorigenesis through the hypomethylation of specific genes, as shown in this research. Further research is needed in these field in CS models [16,18].

In CS, decreased methylation was also observed in cytosine–guanosine dinucleotide (CpG) sites. It was associated with increased expression of the epithelial-specific markers Mammary serine protease inhibitor (Maspin), encoded by the serine protease inhibitor b5 (*SERPINB5*), and 14-3-3σ, encoded by Stratifin (*SFN*), during the development and progression of CS cells after DAC treatment with DAC (Table 1) [19]. Maspin is a regulatory protein that cooperates with a variety of intracellular and extracellular proteins and regulates cell adhesion, motility, apoptosis, and angiogenesis [20]. The tissue-specific expression of Maspin is epigenetically controlled and aberrant methylation of the *Maspin* promoter is closely associated with *Maspin* gene silencing [20]. 14-3-3σ expression is regulated by a p53-dependent pathway and by epigenetic deregulation. Moreover, 14-3-3σ is a significant G2/M cell cycle checkpoint regulator and inhibits nuclear localization of the CDC2/cyclin B complex, which is essential for mitosis progression through mitosis [21,22]. *14-3-3σ* was also revealed to be epigenetically silenced in many tumors by methylation of CpG and can cause tumor development and progression through impaired cell cycle control [22,23]. In the same study by Fitzgerald et al. [19], the treatment of chondrocyte cells with DAC led to the downregulation of the transcription factor snail, the mediator of epithelial–mesenchymal transitions (EMT), [16]. These results showed an epigenetic switch associated with the mesenchymal to epithelial transition (MET) in CS [16].

Furthermore, the microenvironment may play a role in global hypomethylation processes in CS. The study by Hamm et al. investigated the impact of the microenvironment on the methylation status of SRC cells [24]. In their study, the researchers transplanted SRC cells into different positions in Sprague-Dawley rats, e.g., subcutaneous and tibia, and performed pyrosequencing to distinguish the methylation status of these locations compared with normal cartilage tissue [24]. The study revealed differences in gene expression profiles in SRC and normal cells. The researchers indicated that thymosin-β4, FBJ Murine Osteosarcoma Viral Oncogene Homolog (c-fos), and connective tissue growth factor (CTGF) may play a role in CS development and metastatic spread [24]. Furthermore, different sites of transplantation had a significant impact on the epigenetic profile of SRC cells. The subcutaneous tumors were larger compared to tibial tumors, but the tibial tumors were more invasive. However, in both tumor types, the genome was hypomethylated compared with normal cartilage tissue. In conclusion, the microenvironment can affect DNA methylation in CS cells; however, there are still limited data in this area and further research is needed [24].

### 2.2. DNA Hypermethylation

DNA methylation is an epigenetic process that leads to the addition of a methyl (CH_3_) group to a CpG in the DNA chain. This mechanism alters the silencing of DNA activity and gene expression [25]. DNA hypermethylation has been confirmed to play an important role in the pathogenesis of multiple tumor types, including lung, breast, liver, and colon cancer, as well as melanoma, and glioma [26,27].

Multiple genes are involved in this process, including the isocitrate dehydrogenase 1 (*IDH1*)/isocitrate dehydrogenase 2 (*IDH2*) genes, which encode cytosolic (IDH1, NADP+) and mitochondrial (IDH2, NADP) enzymes. These genes are found to be altered in acute myeloid leukemia [28], glioma [29], cholangiocarcinoma [30] and CS [31]. *IDH1* mutations are commonly found in CS—in approximately 50% of all CSs [31]—and have major impacts on cell metabolism and proliferation. In normal cells, IDH1/IDH2 plays an important role in the tricarboxylic acid cycle by isocitrate to α-ketoglutarate conversion [32]. The mutated *IDH*—with a gain of novel catalytic activity—promotes the accumulation of δ-2-hydroxyglutarate (D2HG), an oncometabolite, which causes inhibition of α-KG-dependent dioxygenases and, consequently, hypermethylation of DNA and histones [33]. Consequently, many important biological functions are blocked, e.g., regulation of DNA hydroxymethylation, RNA and histone demethylation, and prolyl hydroxylation of collagen and hypoxia-inducible factors [34]. Disturbances in these mechanisms can lead to overexpression of oncogenic genes or underexpression of tumor suppressor genes and, consequently, progress to malignancy [34].

The study conducted by Guilhamon et al. [35] has demonstrated independent activation of the retinoic acid receptor (RAR) signaling pathway in primary CS with the *IDH* mutation-correlated hypermethylation phenotype. The authors suggested that inhibition of ten-eleven-translocation methylcytosine dioxygenase (TET) enzymes could be a mechanism of DNA hypermethylation in CS with *IDH* mutations. TET enzymes are inhibited by the increased production of D2HG. This process affects the methylation of DNA at CpG islands, which then enrich for genes associated with stem cell maintenance, differentiation, and lineage specification [36,37]. On the other hand, the rare occurrence of *TET* mutations simultaneously with *IDH* mutations suggests another mechanism of this event [35,38]. The TET enzymes work through the oxidation of 5-methylcytosines (5-mC), which results in a decrease in DNA methylation [39]. The study by Lu et al. [37] showed that *IDH* mutations were also related to DNA hypermethylation at CpG islands in CS. Another study demonstrated that levels of 5-mC and 5-hydroxymethylcytosine (5-hmC) in central CS were variable, but not associated with *IDH* mutations and not directly dependent on TET inhibition by 2HG [40]. In addition to this, studies on human CS cells with the inhibitor of IDH1—N-[2-(cyclohexylamino)-1-(2-methylphenyl)-2-oxoethyl]-N-(3-fluorophenyl)-2-methyl-1H-imidazole-1-acetamide (AGI-5198) decreased D2HG levels in two cell lines and inhibited cell formation and migration, with interruption of cell cycling and apoptosis induction [41]. However, the study by Suijker et al. [42] with the AGI-5198 agent—the first highly potent and selective inhibitor of IDH1 R132H/R132C mutants—showed that the use of mutated IDH inhibitors may not always be efficient for the treatment of operable or metastasized CS patients [42]. In another study, almost all primary cartilage tumors with a mutation in *IDH* presented the CpG island methylator phenotype (CIMP). The tumors were clustered into two groups: benign/low-grade tumors and high-grade tumors [43]. The analysis performed in this study demonstrated that grade 3 CSs were more strongly methylated than grade 2 CSs. Furthermore, due to promoter methylation, both signal transduction and inflammation-related genes were affected [43]. In the same study, an epigenetic compound screen was performed to indicate potential novel targets for the therapeutic strategy. The results showed that inhibitors of several proteins, i.e., Aurora kinase inhibitors, bromodomain and extra-terminal motif (BET), Fms related receptor tyrosine kinase 3 (FLT3), histone deacetylase (HDAC) inhibitors, and Janus kinase (JAK), reduced the growth of all CS cell lines (independent of the *IDH* mutations status) [43].

Hypermethylation of CpG islands was also observed in dedifferentiated CS. In this study, the low-grade chondroid compartment presented hypermethylation of *p16^INK4^* and E-cadherin (*CDH1*) promoters (Table 1) [44]. The osteosarcomatous compartment of dedifferentiated CS has the same aberrations in *p16^INK4^*, fragile histidine triad diadenosine triphosphatase (*FHIT*) (Table 1) and *CDH1* promoters. No methylation in *p14^ARF^* and *p21^WAF1^* promoters was detected [44]. Other genes involved in the hypermethylation of the CpG islands in CS are nicotinamide phosphoribosyl transferase (*NAMPT*) and nicotinic acid phosphoribosyl transferase (*NAPRT*)—intracellular enzymes that catalyze the first step in the biosynthesis of NAD from nicotinamide and nicotinic acid [45]. Hypermethylation in the promoters of these genes led to decreased cell viability. However, the pro-apoptotic effect on CS cell lines was not related to the status of the *IDH* mutation. Moreover, an increased level of methylation of the *NAPRT* promoter was observed in high-grade, compared with low-grade, CS. It suggests that NAMPT and NAPRT inhibitors may be potentially useful in the treatment of high-grade CS [45].

Diminished expression of the RUNX family transcription factor 3 **(***RUNX3*) tumor suppressor gene is found in many cancers, for example, breast cancer [46] or hepatocellular carcinoma [47]. Jin et al. [48] confirmed that this gene is also underexpressed in CS. The molecular analysis of tumor specimens acquired from patients who did not undergo chemotherapy or radiotherapy revealed that RUNX3 protein and *RUNX3* transcript levels were reduced compared with normal tissue. Immunostaining of tissue samples also showed loss of RUNX3 expression relative to normal tissue [48]. The methylation status of CS cells assessed by methylation-specific PCR (MSP) confirmed excessive methylation of the *RUNX3* promoter region. RUNX3 expression was strongly associated with a positive prognosis in patients with CS. The same research showed that a poor prognosis was correlated with a negative expression of RUNX3. To verify the results, the CS cell line SW135 was transfected with pcDNA3.1-RUNX3. The results similarly revealed that RUNX3 expression inhibits proliferation and promotes apoptosis in CS cells [48]. Another study demonstrated that Apolipoprotein B MRNA Editing Enzyme Catalytic Subunit 3B (APOBEC3B) caused a reduction in the antitumor activity of RUNX3. APOBEC3B protects the immune system from retrovirus infection and protects the cell from endogenic mobile retroelements [49,50]. Cells without *APOBEC3B* knockdown had a lower apoptotic ratio than RUNX3-positive SW1353 cells with *APOBEC3B* knockdown, so it could be deduced that APOBEC3B obstructs *RUNX3* transcription. Potential therapy to improve apoptosis in CS may involve the suppression of *APOBEC3B* knockdown [50].

There is an association between the expression of the p73 protein and aberrant methylation patterns in CS [51]. p73 is a protein which is part of the p53 tumor suppressor family. Due to their similar structures, both p53 and p73 are regarded as anti–oncogenic factors [52,53]. p73 shows the ability to influence the transcription of genes controlled by p53; for example, *p21^WAF1^*, mouse double minute 2 homolog (*MDM2*), B cell CLL/lymphoma2 (Bcl-2) Associated X-protein (*BAX*), *14-3-3s*, and phorbol-12-myristate-13-acetate-induced protein 1 (*PMAIP1*, *NOXA*) [51,54]. As a result, the cell undergoes apoptosis or cell cycle arrest [55,56]. According to Liu et al. [51], in CS cell lines, the expression of p73 was significantly decreased as a result of hypermethylation of the *p73* promoter region. Furthermore, the level of methylation correlated with the histological grade of the tumor. Grade 2 and 3 CS cells had a markedly higher level of methylation compared to grade 1 CS. Therefore, the analysis of methylation could be used as a prognostic tool and p73 could be a target for the treatment of CS. The research conducted by Tan et al. [57] revealed that pigment epithelium-derived factor (PEDF) induced apoptosis in the CS cell line. The molecular analysis has shown changes in the expression of multiple factors that participate in the cell cycle and apoptosis. Importantly, p73 expression was significantly elevated. PEDF works through multiple mechanisms as it affects molecules involved in apoptosis, cell adhesion, and invasion of cells. Therefore, it should be investigated as a candidate for the targeted treatment of CS [57].

**Table 1:** Genes hypomethylated and hypermethylated in chondrosarcoma.

Gene or DNA Region Name	Function in Normal Cells	Effect in Chondrosarcoma	Reference
Genes Hypomethylated in Chondrosarcoma			
Satellite 1	Maintenance of chromosome structure.	Increased proliferation, ability to metastasize	[16,58]
L1	Determination of transposition—converting RNA into DNA to insert themselves into different genomic locations (called transposons). This gene becomes inhibited in somatic cells and activated in germline cells and embryogenesis.	Initiation of cancer and progression to malignancy	[10,15]
*SERPINB5*	Regulation of cell adhesion, motility, apoptosis, angiogenesis.	Progression to malignancy	[19,20]
*SFN*	Effect on genetic, molecular, and cellular levels of inflammation. Impact on cell proliferation and differentiation.	Progression to malignancy	[10,19,59]
*MDK*	Embryogenesis, fetal development, organogenesis, neurogenesis, epithelial–mesenchymal interactions.	Progression to malignancy	[16,60]
*SOX2*	A pluripotent growth factor important in embryonic development and which plays a fundamental role in maintaining embryonic cell stemness and various adult stem cell populations.	Progression to malignancy	[10,16,61]
Genes hypermethylated in chondrosarcoma			
*p16^INK4a^*	Tumor suppressor encoding the inhibitor of CDK4/6.	Cell cycle progression, increased proliferation	[62,63]
*RUNX3*	Forms a complex with pRb, Brd2 and induces p21 protein which stops progression to phase S. Recruitment of Trithorax and Polycomb complexes and regulates the structure of chromatin, which decides whether the cell can go through the R-point.	Increased proliferation, reduced apoptosis	[48,64,65]
*FHIT*	The tumor suppressor gene owns pro-apoptotic abilities (activating caspases 3, 8, and 9), keeps up genome integrity.	Induced carcinogen transformation	[66]
*CDH1*	An important role in cell proliferation, cell adhesion, cell polarity, and in epithelial–mesenchymal transition.	The proliferation of tumors, invasion, migration and metastasizing	[10,44,67]
*3-OST-2*	Production of heparan sulphate proteoglycans which regulate cell adhesion, proliferation, and interactions with molecules such as growth factors or cytokines.	Increased proliferation and invasiveness of chondrosarcoma cells	[17,68]
*p73*	Induction of apoptosis, cell cycle arrest.	Progression to malignancy	[51,54]

Abbreviations: Bromodomain containing 2 (Brd2); Cyclin-dependent kinases (CDK); E-cadherin gene (CDH1); fragile histidine triad diadenosine triphosphatase (FHIT); heparan sulfate D-glucosaminyl 3-O-sulfotransferase (3-OST-2); long interspersed nuclear elements 1 (LINE-1; L1); neurite outgrowth-promoting factor 2 (midkine, MDK); Retinoblastoma protein (pRb); RUNX family transcription factor 3 (RUNX3); Serine protease inhibitor b5 (SERPINB5); Stratifin (SFN); SRY-related HMG-box transcription factor 2(SOX2).

## 3. MicroRNAs (miRNAs)

MicroRNAs (miRNAs) are small noncoding RNAs involved in the post-transcriptional regulation of gene expression and, as epigenetic modulators, affect the protein levels of the target mRNAs without modifying the gene sequences [17,69]. They are mainly endogenous and transcribed from genomic DNA. By binding to 3′UTRs and inducing transcript degradation or translational repression, miRNAs can induce the downregulation of their target mRNAs. However, miRNAs can also bind to other regions within the mRNAs, which, in certain situations, induces gene expression upregulation [70,71]. MicroRNAs are involved in chondrogenesis and cartilage diseases [72], and they may also act as tumor suppressors or as oncogenes [73]. There are several thousand miRNAs in humans [74]. MicroRNA can control angiogenesis by inhibiting vascular endothelial growth factor A (VEGF-A) signaling and CS cell proliferation [75]. Two important main studies, which revealed down-regulation of let-7a, hsa-miR-100, hsa-miR-136, hsa-miR-222, hsa-miR-335, and hsa-miR-376a, and up-regulation of hsa-miR-96 and 183 in CS cell lines and tissue samples, were published by Yoshitaka et al. [76] and Nugent et al. [77]. The most important miRNAs in CS are, among others, hsa-miR-30a, which inhibits proliferation; hsa-miR-218 and hsa-miR-524-5p, which increase proliferation; hsa-miR-125b and hsa-miR-192, which enhance  chemosensitivity; hsa-miR-16-5p, which promotes  angiogenesis; hsa-miR-519d and hsa-miR-145, which inhibit metastases; and hsa-miR-26a and hsa-miR-199a, which inhibit angiogenesis [78]. Liang et al. [79] have shown that hsa-miR518b overexpression decreases the expression of Bcl-2 in human CS cell lines, which induces apoptosis and inhibits cell migration.

On the other hand, hypoxia regulates hsa-miR-181 in CS, which up-regulates the expression of VEGF and matrix metalloproteinases (MMPs) [80]. Overexpression of hsa-miR-181a was observed in high-grade CS, promoting tumor progression. In 2019, Sun et al. [80] published results of the systemic and local intratumoral use of anti-miRNA oligonucleotides directed against hsa-miR-181a, the regulator of G-protein signaling 16 (RGS16) [80,81]. In general, the removal of hsa-miR-181a restored RGS16 expression and inhibited tumor progression [80]. Hameetman et al. [82] also found that miRNAs are involved in the malignant transformation of osteochondroma to CS.

Galoian et al. [83] have found up-regulation of tumor suppressors hsa-miR-20a, hsa-miR-125b, hsa-miR-192, and down-regulation of onco-miRNAs, hsa-miR-490-3p, hsa-miR-509-3p, hsa-miR-589, and hsa-miR-550 in the human JJ012 CS cell line treated with the mammalian target of the rapamycin complex 1 inhibitor (mTORC1). In another study carried out in CS cells and in vivo in mice, overexpression of breast cancer anti-estrogen resistance 4 (BCAR4), a long noncoding RNA (lncRNA) that participates in the formation of multiple cancers [84], resulted in hyperacetylation of histone H3 in the mammalian target of rapamycin (*MTOR*) promoter. Activation of the mTOR signaling pathway led to the progression of CS cells through the proliferation and migration of CS cells [73]. In vivo experiments confirmed that increased tumor growth was associated with BCAR4 overexpression. On the other hand, blocking this pathway nullified the outcome of BCAR4 expression [73].

Nicolle et al. analyzed a series of 102 cartilage tumors, mostly CSs (89%), from 8 clinical sites in France treated between 1997 and 2013 [85]. Among many parameters evaluated, CS microRNA profiling was performed using RNAseq. The most differentially expressed microRNAs were frequently found at the 14q32 locus, defining the level of malignancy. Therefore, assessment of miRNA could be used as a prognostic tool in CS.

MiRNAs may also be involved in mechanisms responsible for CS resistance to chemotherapy. Tang et al. [86] discovered that hsa-miRNA-125b causes sensitization of CS cell to treatment with doxorubicin via inhibition of erb-b2 receptor tyrosine kinase 2 (ErbB2) and glucose metabolism. ErbB2 is known to be overexpressed in cancer cells and promote glycolysis and further proliferation of cells [87]. These findings show a new possible approach to treatment of CS with a combination of epigenetic drugs and chemotherapy.

## 4. Post-Translational Modification of the Histones

### 4.1. Acetylation

Acetylation of histones is a process that leads to the addition of an acetyl group to lysine residues present in the core histones. As a result, the ionic charge of histone changes from positive to neutral. DNA, which has a negative charge, becomes separated from histones and, consequently, transcription factors gain access to DNA. Therefore, hyperacetylation is associated with the active expression of genes. HDAC are enzymes that decrease histone acetylation, resulting in chromatin remodeling and decreased expression of particular genes. [88] In tumors, down-regulated expression is related to proteins involved in the cell cycle and proliferation. Several experiments revealed that HDAC inhibitors can restore abnormal gene silencing and stop tumor progression [88,89]. Therefore, they can be a potential target for the treatment of CS.

One of the candidates in CS treatment, targeting HDAC, may be resveratrol, which activates the expression of Sirtuin1 (*SIRT1*). Abnormal *SIRT1* expression increases the metastatic potential of CS cells by induction of the EMT [90]. Additionally, SIRT1 expression was correlated with tumor progression and prognosis in patients with CS [90]. Resveratrol acts as an nuclear factor kappa-light-chain-enhancer of activated B cells (NF-κB) deactivator through deacetylation of the p65 subunit, which forms NF-κB. It also promotes apoptosis through the activation of caspase 3 [91]. The antiapoptotic ability of resveratrol was confirmed in vivo by using a CS cell xenograft in mice. Tumor growth after resveratrol treatment was significantly inhibited. After tumor excision, SIRT1 and caspase 3 levels in tumor cells increased [91].

Another HDAC inhibitor, depsipeptide, a protein that promotes the expression of the p21 protein and regulates the cell cycle [92], was observed to induce apoptosis and cell cycle arrest in CS cells [93]. Furthermore, the expression of the collagen alpha-1(II) chain (*COL2A1*) gene was also increased as a consequence of histone H3 acetylation in the promoter and enhancer of *COL2A1*. The depsipeptide also has an impact on the composition of the extracellular space due to higher levels of aggrecan expression and a2 chain of type XI collagen [93]. HDAC inhibitors can also regulate CS cell differentiation. Histological analysis has shown that CS cells treated with depsipeptide differentiate into the hypertrophic phenotype [93]. These antitumor effects were also confirmed in vivo by decreased tumor growth and markedly more differentiated cells.

Inhibitors of deacetylases can also regulate CS cell proliferation by a mechanism that is not related to epigenetics. Histone deacetylase 6 (HDAC6) can regulate the structure of the cytoskeleton and cilia [94,95]. CS lacks the proper sensor of primary cilia due to the increased activity of HDAC6. Inhibition of HDAC6 with tubastatin A caused inhibition of CS cell proliferation. Increased expression of acetylated a-tubulin, a protein present in the cilia, was also observed in the affected cells [96].

### 4.2. Methylation

Histone methylation plays a significant role in gene expression. This process is carried out by two classes of enzymes: histone methyltransferases (HMTs) and histone demethylases (HDMs). Depending on the position of methylation, it can promote or suppress the transcription of genes [97]. For example, methylation of histone H3 lysine K4 (H3K4), histone H3 lysine K36 (H3K36), and histone H3 lysine K79 (H3K79) promotes transcription, while methylation of histone H3 lysine K9 (H3K9), histone H3 lysine K27 (H3K27), and histone H4 lysine K20 (H4K20) leads to repression of transcription by chromatin remodeling [97]. H3K4, H3K9, and H3K27 are believed to control the promoter region of SRY-Box transcription factor 9 (*SOX9)* and *COL2A1* genes [98,99]. HDM inhibitors have already been shown to decrease cell proliferation in gliomas and acute lymphoblastic leukemia [100,101]. Due to the positive results of research conducted on different tumors, HDMs have begun to be investigated in CS [102].

One of the HDM inhibitors studied in CS cells is GSK-J4, which has shown an antiproliferative effect on CS cells [103]. GSK-J4 is an inhibitor of histone demethylases of Lysine specific demethylase 6A (KDM6A, UTX) and Lysine demethylase 6B (KDM6B, JMJD3). Both UTX and JMJD3 regulate the methylation of H3K27. GSK-J4 has been proven to reduce proliferation in CS without affecting normal chondrocytes. Moreover, it induces apoptosis and senescence in CS cells. The combination of GSK-J4 and cisplatin showed a decreased proliferation of CS cells compared with treatment with cisplatin or GSK-J4 alone, but not all CS cell lines were treated with both drugs. Due to the insufficient amount of data, more molecular tests should be performed before moving to pharmacological treatment [103].

Another HDM described in CS is lysine-specific demethylase 1 (LSD1). This enzyme participates in stem cell proliferation and differentiation, as well as in the regulation of EMT via repression of *CDH1* [104]. LSD1 demethylates H3K4 mono-/dimethylation (H3K4me1/2), leading to the repression of gene expression. At the same time, it induces H3K9me1/2 demethylation, which activates the expression of genes [105,106]. Its influence on epigenetics suggests that it is a proto-oncogenic factor. LSD1 is overexpressed in CS, similarly to other tumors, e.g., neuroblastomas, breast carcinomas, leukemias, bladder, lung, colorectal carcinomas, and other types of sarcomas [107,108]. Therefore, it may be another promising target to investigate. A candidate drug can be Tranylcypromine, which is an inhibitor of monoamine oxidase used to treat patients with depression and anxiety [109]. On the other hand, it also has the effect of irreversibly inhibiting LSD1. Tranylcypromine has shown an antiproliferative effect in neuroblastoma, breast carcinoma, and synovial sarcoma, supporting further research on LSD1-targeting drugs in the treatment of CS [108].

One more therapeutic possibility for CS involves proline-rich polypeptide-1 (PRP-1). It is an inhibitor of H3K9 demethylase that can restore the expression of anti-inflammatory cytokines and has the potential to be used as an antiproliferative agent [110]. PRP-1 can reestablish the expression of suppressor of cytokine signaling 3 (SOCS3) and ten-eleven-translocation methylcytosine dioxygenase 1 and 2 (TET1/2) [111,112]. SOCS3 is considered to be responsible for suppressing pro-inflammatory factors [113]. The inactivation of *SOCS3* and *TET1*/*2* was caused by the demethylation of histone H3K9 in the promotor regions of these proteins [114,115]. PRP-1 restored the proper level of methylation in these promotor regions. As a consequence, the population of stem cells from CS decreased. The ability of PRP-1 to restore the normal level of expression of anti-inflammatory cytokines and reduce tumor growth makes it a potential therapeutic agent [116].

The enhancer of zeste homolog 2 (EZH2) is part of the polycomb repressive complex 2 (PRC2), which has HMT activity and is responsible for H3K27 methylation, leading to a decrease in gene transcription [117,118]. The study by Girard et al. [119] showed that the EZH2 protein was expressed in CS, whereas it was not present in enchondromas or chondrocytes, suggesting the role of this protein in the pathogenesis of CS. Furthermore, the level of EZH2 expression correlated with the grade of CS and could potentially be used as a prognostic factor [119]. The study also revealed that EZH2 expression can be reduced by 3-Deazaneplanocin (DZNep), an inhibitor of S-adenosyl homocysteine hydrolase (SAH) inhibitor [119]. Using DZNep in vitro led to a depletion of EZH2 expression and, consequently, loss of methylation of H3K27 [119,120]. In many studies, DZNep demonstrated the ability to inhibit tumor growth, e.g., in breast and hepatocellular cancer in vitro [121,122]. In vitro studies revealed that DZNep inhibited tumor growth and migration and promoted apoptosis in CS cells with the down-regulated activity of *EZH2*. Interestingly, DZNep slightly reduced the growth of normal chondrocytes [119]. However, its antiproliferative effect may not only be related to the inhibition of *EZH2*. DZNep is not selective and inhibits methylation globally, so its effect on CS cells is more complicated and requires further examination [119]. The next study conducted by Lhuissier et al. [123] showed that the combination of DZNep and cisplatin was more effective in reducing CS cell growth in comparison to the use of each of these drugs alone. This study presents a potentially new way of treating CS with epigenetic drugs and standard chemotherapy.

In CS, the methylation of H3K4, H3K9, and H3K27 appears to be independent of the mutation of the *IDH1/2* gene, contrary to what is observed in other tumors such as gliomas [40,42]. In the study by Suijker et al. [42], both the DNA methylation pattern and the methylation of H3K4, H3K9, and H3K27 were not altered after treatment of CS cells with the AGI-5198 [40,42]. However, these histone modifications seem to be relevant only in the formation of enchondromas; therefore, in CSs, it is necessary to search for other processes causing modifications of histones [98,99].

However, other studies indicate that loss of methylation in H3K27 can affect the clinical and histopathological features of CS [124]. This was already described in dedifferentiated CS, which is considered to be related to mutations in the Embryonic Ectoderm Development (*EED)* and the suppressor of the Zeste 12 Protein Homolog (*SUZ12)* genes, belonging to PRC2. On the other hand, these genes were not mutated in well-differentiated CS [124]. Therefore, changes in the activity of PRC2 may play a significant role in the dedifferentiation process of CS. The histological characteristics presented by dedifferentiated CS with histone modification resemble the malignant peripheral nerve sheath tumor (MPNST) with spindle cells [125]. These two tumors can be distinguished by analysis of the Neurofibromatosis type 1 (*NF1*) mutation, which typically occurs in nerve sheath tumors, and by the presence of *IDH2, COL2A1, SUZ12* or *EED* mutations, related to CS [124].

**Figure 1:**
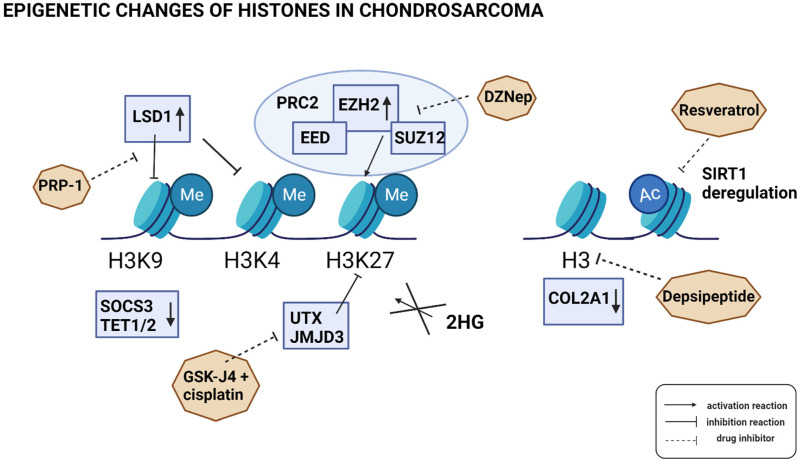
Epigenetic changes to histones in chondrosarcoma (CS). The mechanism of histone modification is still not fully understood; however, some proteins were observed to play a role in this process in CS. The methylation of histone H3 lysine K9 (H3H9), histone H3 lysine K4 (H3K4), and histone H3 lysine K27 (H3K27) appears to be independent of δ-2-hydroxyglutarate (2HG); however, the methylation of H3K27 is related to increased expression of enhancer of zeste homolog 2 (EZH2)—a part of the Polycomb Repressive Complex 2 (PRC2), together with Embryonic Ectoderm Development (EED) and Suppressor of Zeste 12 Protein Homolog (SUZ12). This process can be inhibited by 3-Deazaneplanocin A (DZNep). On the other hand, H3K4 and H3K9 demethylation (which leads to inactivation of the suppressor of cytokine signaling 3 (*SOCS3*) and ten-eleven translocation methylcytosine dioxygenase 1 and 2 (*TET1/2*)), is caused by the expression of histone lysine-specific demethylase 1 (LSD1). This can be inhibited by proline-rich polypeptide-1 (PRP-1). Lysine-specific demethylase 6A (KDM6A, UTX) and lysine demethylase 6B (KDM6B, JMJD3) decrease H3K27 methylation, which can be blocked by the GSK-J4 inhibitor in combination with cisplatin. Sirtuin1 (SIRT1) deacetylase deregulation can be blocked by resveratrol, while decreased acetylation of H3 and collagen alpha-1(II) chain (*COL2A1*) expression can be reversed by depsipeptide.

To summarize, the analysis of histone modifications could be useful for the diagnosis of CS and the introduction of specific drugs; however, further studies are needed to explain the precise mechanism of these changes. A summary of histone modifications and potential therapeutic drug candidates already found in CS is presented in Figure 1.

## 5. SUMOylation

Small ubiquitin-like modifier (SUMO) proteins constitute a group of proteins which participate in post-translational modifications of molecules [126]. The study conducted by Kroonen et al. [127] revealed that expression of SUMO in CS is elevated. Moreover, increased expression of SUMO 1 and SUMO 2/3 corresponded with higher histological grade and a higher level of SUMO 2/3 resulted in worse overall survival. Therefore SUMO 2/3 has the potential to become a prognostic marker. In vitro studies performed in the same research showed that inhibition of SUMO E1 reduced CS cell proliferation and viability. Dedifferentiated CS cell lines, which are known for aggressiveness, were exceptionally susceptible to inhibition of SUMO E1. To summarize, introduction of drugs targeting SUMO may lead to more effective treatment of CS [127].

## 6. Therapies against Epigenetic Modifications

Epigenetic modifications, based on promising preclinical data, should be the area of extended research and development in CS. Most potential drugs are currently being evaluated for safety and dosage in phase 1 clinical trials in solid tumors and hematological malignancies. Some limited studies are specifically enrolling patients with CS, but most of the studies enrolled only participants with solid tumors to assess doses, safety and preliminary efficacy signals. Examples of studies conducted in CS as well as in the general population of solid tumors have been summarized in Table 2.

Studies concerning IDH inhibitors have also included patients with CS. Currently, there are three ongoing clinical trials which use drugs targeting the mutant IDH1. The drugs that are being tested are LY3410738 (NCT04521686) and Ivosidenib (AG-120) (NCT04278781, NCT02073994). As mentioned above, *IDH1* mutations are common in CS and these substances have the potential to inhibit CS growth. The results of the research using AG-120, administered orally in advanced CSs, showed that the level of plasma D2HG decreased in all patients, and in half of the patients (52%, 11 out of 21), most tumors stopped growing [128]. Currently, another phase II trial study (NCT04340843) has been started on patients with unresectable and metastatic CSs treated with the HDAC inhibitor belinostat in combination with hypomethylating agents (SGI-110 (guadecitabine) or ASTX727 (cedazuridine)). However, this has been suspended due to the pending completion of the safety lead-in [129].

Besides IDH inhibitors, other drugs have been tested, such as LSD1 and BET inhibitors (NCT03895684, NCT02419417). Dose and safety studies with these two drugs on solid tumors have been completed. Preliminary efficacy data of a phase 1/2a, open-label study with BMS-986158 monotherapy (NCT02419417) revealed stable disease (SD) in 26.1% to 37.5% of patients, depending on dosing schedules. BMS-986158 was well tolerated in patients with schedule A dosing (5 days on, 2 days off) and its antitumor activity was noted by 30.4% of these patients [130]. Currently, one more phase 1b/2 study, including patients with advanced or metastatic solid tumors treated with TAK-981 (SUMO inhibitor) in combination with pembrolizumab, is ongoing (NCT04381650).

## 7. Conclusions

During the last decade, our knowledge about epigenetic alterations and their influence on the pathogenesis of cancers has changed significantly. Due to the poor effectiveness of chemotherapy and radiotherapy in the treatment of CS, there is a constant need for the development of new therapies. In the future, epigenetic changes may be used as a potential prognostic and predictive factor as well as a therapeutic target in CS treatment. It is known that modification of DNA and histones, such as methylation or acetylation, as well as miRNAs, can play an important role in the pathogenesis of CS. Examining these modifications gives us valuable insight into the pathogenesis of tumors. However, this is particularly challenging, since the exact mechanisms of the processes involved in epigenetic alterations are still not fully understood. MicroRNAs and SUMO have the potential to be used as prognostic factors. Abnormalities in the expression of these molecules can indicate sensitivity to chemotherapy in CS as well as be an indicator of prognosis and overall survival. Analysis of the methylation level of genes related to the pathogenesis of CS could be used to diagnose and assess its histological and clinical characteristics. So far, none of the drugs that influence epigenetic changes have been widely accepted in treatment and there are only a few potential candidates to implement. However, epigenetic alterations in CS should be the subject of intensive research in upcoming years.

## Figures and Tables

**Table 2:** Ongoing clinical trials for chondrosarcoma (https://clinicaltrials.gov, accessed on 22 December 2022).

ClinicalTrials.Gov Identifier	Study Title	Conditions	Mechanism of Action
NCT04521686	Study of LY3410738 Administered to Patients with Advanced Solid Tumors with IDH1 or IDH2 Mutations	Cholangiocarcinoma Chondrosarcoma Glioma Any Solid Tumor	Inhibitor of isocitrate dehydrogenase 1 (IDH1)
NCT04278781	AG-120 (Ivosidenib) in People with IDH1 Mutant CS	Chondrosarcoma, Grade 2 Chondrosarcoma, Grade 3 IDH1 Gene Mutation	Inhibitor of IDH1
NCT02073994	AG-120 in Subjects with Advanced Solid Tumors, Including Glioma, With an IDH1 Mutation	Cholangiocarcinoma Chondrosarcoma Glioma Other Advanced Solid Tumors	Inhibitor of IDH1
NCT03895684	Phase 1 Trial of the LSD1 Inhibitor SP-2577 (Seclidemstat) in Patients with Advanced Solid Tumors	Solid tumors	Lysine-specific demethylase 1 (LSD1) Inhibitor
NCT02419417	Study of BMS-986158 in Subjects with Select Advanced Cancers (BET)	Solid tumors	Small molecule inhibitor of the bromodomain and extra-terminal (BET) proteins
NCT04381650	A Study of TAK-981 Given with Pembrolizumab in Participants with Select Advanced or Metastatic Solid Tumors	Advanced or Metastatic Solid Tumors	TAK-981—small ubiquitin-like modifier (SUMO) inhibitor
NCT04340843	Testing the Combination of Belinostat and SGI-110 (Guadecitabine) or ASTX727 (Cedazuridine) for the Treatment of Unresectable and Metastatic Conventional Chondrosarcoma	Locally Advanced Unresectable Primary Central Chondrosarcoma Metastatic Primary Central Chondrosarcoma Unresectable Primary Central Chondrosarcoma	Belinostat—histone deacetylase inhibitor Guadecitabine—hypomethylating agent;oral decitabine and cedazuridine—hypomethylating agent

## Data Availability

Not applicable.
